# Polynucleotides HPT‐Based Dermal Filler for Skin Rejuvenation: A Prospective Clinical Investigation

**DOI:** 10.1111/jocd.71154

**Published:** 2026-08-25

**Authors:** Chiara Teramo, Andrea Mattioli, Carolina Prussia

**Affiliations:** ^1^ Department of Integrated Surgical and Diagnostic Sciences (DISC), Plastic and Reconstructive Surgery Clinic University of Genoa Genoa Italy; ^2^ Medical Statistics Consultant Locarno Switzerland

**Keywords:** dermal filler, PN HPT, polynucleotides, skin elasticity, skin hydration, skin rejuvenation, skin turgor

## Abstract

**Background:**

Polynucleotides (PN)‐based dermal fillers are increasingly used for skin rejuvenation due to their biocompatibility and regenerative properties.

**Aims:**

This study aimed to confirm the efficacy and safety of Plinest (Mastelli) in improving skin hydration of the face, neck, and décolleté over 4 months follow‐up.

**Patients/Methods:**

Participants received three injections at 2‐week intervals (at visits V0, V1, and V2), with follow‐up at two (V3) and 4 months (V4) after last injection. The primary outcome was aesthetic improvement measured by the Global Aesthetic Improvement Scale (GAIS), while secondary outcomes included skin hydration, elasticity, turgor, patient satisfaction, and tolerability.

**Results:**

Twenty‐eight subjects were enrolled. GAIS scores showed significant improvement across all areas from baseline to V4, with a median increase of 2 points, as reported by subjects and investigator. At V1, facial skin hydration increased from 48.8% ± 5.9% to 50.4% ± 6.9%, with similar trends in neck and décolleté. A slight decline was observed at V2, followed by a peak at V3 before stabilizing at V4 (face: 49.6% ± 6.2%, neck: 50.6% ± 7.3%, décolleté: 47.4% ± 4.1%). Skin elasticity improved between V0 and V4, with mean differences ranging from 5 to 11 N/m. Pain was moderate, with higher intensity in the face and neck compared to the décolleté. Despite this, participants were highly satisfied, with scores between 4 and 5 at V3 and V4. Adverse events were mild and transient.

**Conclusion:**

These findings confirm the efficacy and safety of the device in improving skin hydration and demonstrate high patient satisfaction.

## 1 Introduction

Skin aging is a common patient concern in aesthetic medical practice, a sector which is witnessing an increasing demand for skin rejuvenation procedures. The aging process is characterized by a progressive deterioration of skin structure and function, leading to visible and functional changes such as reduced elasticity, impaired barrier function, and progressive dehydration. These alterations significantly affect skin appearance and integrity, and can negatively influence an individual's self‐esteem and psychological wellbeing [1].

Among the manifestations of skin aging, dryness is one of the most common features. Exogenous factors, such as environmental stressors, including climate conditions and pollution, also contribute to skin dehydration [2, 3]. At a physiological level, dry skin is associated with disruption of the skin barrier, primarily due to alterations in lipid composition and epidermal differentiation in the stratum corneum (SC). These changes can result in a reduction in natural moisturizing factors [4] and an increase in water loss [5], leading to skin irritation, itchiness, and discomfort for the patient [6].

A number of treatments have been developed to restore skin quality, including minimally invasive techniques such as botulinum toxin injections, dermal fillers, platelet‐rich plasma, and thread lifting [7]. Among injectable treatments for skin rejuvenation, polynucleotides (PN)‐based products are gaining increasing interest because of their biocompatibility, regenerative properties, and favorable safety profile [8].

The use of PNs for skin enhancement is not a novel approach. Indeed, their ability to stimulate collagen production, improve skin elasticity, promote hair growth, and enhance scar appearance by fostering a favorable environment within tissues has been extensively reported in the literature [9, 10, 11, 12, 13]. Furthermore, it has been demonstrated that PNs can improve dermal quality in subjects seeking correction of nasolabial folds, either as a standalone treatment or as a priming agent to enhance the effects of subsequent HA injections [14].

In order to comply with regulatory requirements which require ongoing confirmation of the safety and performance of medical devices throughout their life cycle, the present clinical study has been designed to evaluate the performance of a PN‐based dermal filler indicated for skin rejuvenation in subjects seeking improvement in skin hydration of the face, neck and décolleté, with a follow‐up period of up to 4 months. This contributes to the growing body of evidence on aesthetic procedures, ensuring that commercial products are consistently updated and scientifically validated.

## 2 Materials and Methods

### 2.1 Study Design and Population

This prospective, monocentric, interventional, post‐market clinical investigation aims to confirm the performance and safety profile of a PN HPT‐based dermal filler (Plinest, Mastelli s.r.l.) in improving skin hydration at a 4‐month follow‐up. The device is a single‐use viscoelastic gel containing 20 mg/mL of polynucleotides PN HPT in a 2 mL pre‐filled syringe isolated from the gonads of freshwater trout ( *Oncorhynchus mykiss* ) using the proprietary High Purification Technology (HPT) process developed by Mastelli s.r.l.

The study included adults up to 70 years, of either sex, who required an improvement in skin hydration in a maximum of two areas of the face, or one area of the face and neck or décolleté. The subjects were classified according to Fitzpatrick's skin phototype classification, ranging from I to IV. Participants were enrolled in the study if they had provided written informed consent. Exclusion criteria included pregnancy, allergies to device components, and the presence of concurrent disease within the tested region. The complete list of inclusion and exclusion criteria is available in the Supporting Information (Table S1).

**FIGURE 1:**
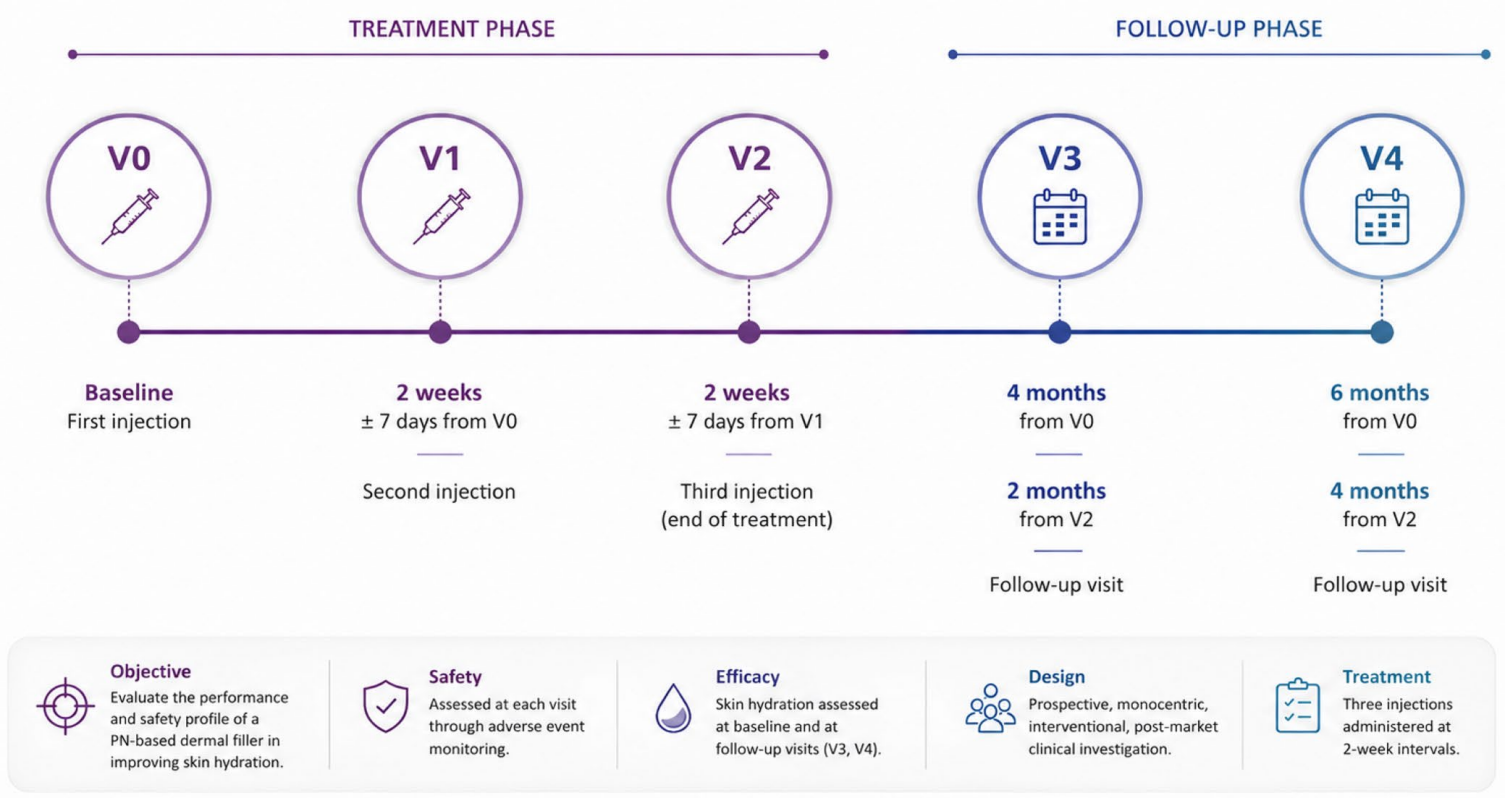
Study design.

Eligible participants were treated with the investigational PN HPT‐base dermal filler. Subjects underwent three injections at two‐week intervals. Local anesthetics containing lidocaine (Emla cream 2.5% + 2.5%) were administered to all participants at each visit. Five visits were planned as described below (Figure 1):

- V0/baseline: first injection
- V1: 2 weeks ±7 days from V0, second injection
- V2: 2 weeks ±7 days from V1, third injection (end of treatment)
- V3: follow‐up visit at 4 months from V0 and 2 months from V2
- V4: follow‐up visit at 6 months from V0 and 4 months from V2.

### 2.2 Study Objectives

The primary objective was to assess the performance of PN HPT‐based dermal filler in improving the aesthetic appearance of the skin 4 months after treatment. To achieve this objective, the primary endpoint was defined as the change in the Global Aesthetic Improvement Scale (GAIS) from baseline to V4, assessed by both participants and the investigator.

Secondary objectives included a more extensive evaluation of product performance in terms of aesthetic improvement, skin hydration, elasticity, and turgor, as well as patient satisfaction and tolerability. The secondary endpoints were defined as changes from baseline at each visit in the GAIS score (a 5‐point scale where 1 = ‘very much improved’, 2 = ‘much improved’, 3 = ‘improved’, 4 = ‘no change’, and 5 = ‘worse’; baseline GAIS score was standardized to a value of 4 for all treated areas and participants), tissue water content (0%–100%, measured using a MoistureMeter EpiD by Delfin Technologies Ltd), as well as the Instant Skin Elasticity—ISE—index (measured before injections using an ElastiMeter by Delfin Technologies Ltd); moreover, a 5‐point Likert scale (with scores ranging from 1 = very poor to 5 = excellent) was used by the investigator to evaluate skin turgor at each visit. The pain intensity was measured after each injection (V0, V1 and V2) using a Numerical Rating Scale (NRS), where 0 indicates no pain and 10 the worst possible pain, collected at participant level, rather than at the treated area level. In a similar way, the assessment of overall satisfaction was conducted at participant level at V3 and V4, employing a 5‐point Likert scale (ranging from 1 = extremely dissatisfied to 5 = extremely satisfied).

The safety profile of the procedure was monitored throughout the investigation. This included evaluating the rate of adverse events (AEs) and device deficiencies.

### 2.3 Ethical Considerations

The study was conducted in accordance with the Declaration of Helsinki and approved by the local Ethics Committee (Comitato Etico Territoriale Liguria, protocol number 316/2024–DB id 13 994) in October 2024. All participants provided written informed consent for their involvement in the present investigation.

### 2.4 Statistical Methods

Descriptive statistics were used for data analysis. Continuous variables were analyzed using the mean, median, standard deviation, minimum and maximum, while categorical variables were analyzed using frequency distributions. Normality was assessed using the Kolmogorov–Smirnov test. For the primary endpoint, the median change in GAIS scores between V0 and V4 was computed for clinician‐ and patient‐reported evaluations separately. Given the non‐normal distribution of GAIS scores, the Wilcoxon signed‐rank test was employed. A *p*‐value of less than 0.05 was considered to be statistically significant, indicating that the median improvement differed significantly from zero. Secondary outcomes were reported descriptively at each visit.

A post hoc statistical analysis was conducted to evaluate the inter‐participant variations between V4 and V0 in terms of skin hydration and skin elasticity. The paired‐samples *t*‐test was utilized to evaluate the mean change over time. Statistical significance was defined as a two‐tailed *p*‐value less than 0.05.

### 2.5 Subjects Disposition

Participants who received at least one study product injection regardless of any protocol deviations were included in the Safety Analysis Set (SAS). The performance analysis was conducted on the Full Analysis Set (FAS) population, comprising all participants who received the study device and had at least one post‐baseline assessment. As no major protocol deviations were identified among the FAS participants, the Per Protocol Set (PPS) is identical to the FAS. The results reported in the manuscript pertain exclusively to the FAS.

## 3 Results

### 3.1 Characteristics of the Cohort

The investigation was conducted on 28 subjects, consisting of 26 women and 2 men. The mean age of the participants was 43.8 ± 14.8 years. Most cases presented with Fitzpatrick Skin Phototype II (*n* = 26; 92.9%). Eleven participants (39.3%) were under concomitant medications, and 10 women were in menopause at the time of enrolment (35.7%). Detailed description is provided in Table 1.

**TABLE 1:** Demographics.

Participants (tot)	28 (100)
Sex, female	26 (92.9)
Age (years)	43.8 (14.8); 21–68
Ethnicity	
Caucasian	26 (92.9)
Hispanic	1 (3.6)
Arabic	1 (3.6)
Fitzpatrick Skin Phototype	
II	26 (92.9)
III	2 (7.1)
Concomitant medication, yes	11 (39.3)
Medical history	
Anxiety	1 (3.6)
Asthma	1 (3.6)
Autoimmune thyroiditis	1 (3.6)
Depression	1 (3.6)
Hypertension	4 (14.3)
Hysterectomy	1 (3.6)
Insomnia	1 (3.6)
Menopause	10 (35.7)
Thalassemia (alpha and beta)	2 (7.1)

*Note:* Data are reported as *n* (%), except for age, which is described as mean (SD); Min‐Max.

Abbreviation: SD, standard deviation.

Among the 28 participants enrolled, 26 completed the first scheduled visit (V1), thus being categorized within the FAS population. One subject was excluded due to a pre‐existing violation of the eligibility criteria, while another withdrew from the study for personal reasons (Figure S1).

Details on the treated areas are presented in the Table S2.

### 3.2 Performance Outcomes

**FIGURE 2:**
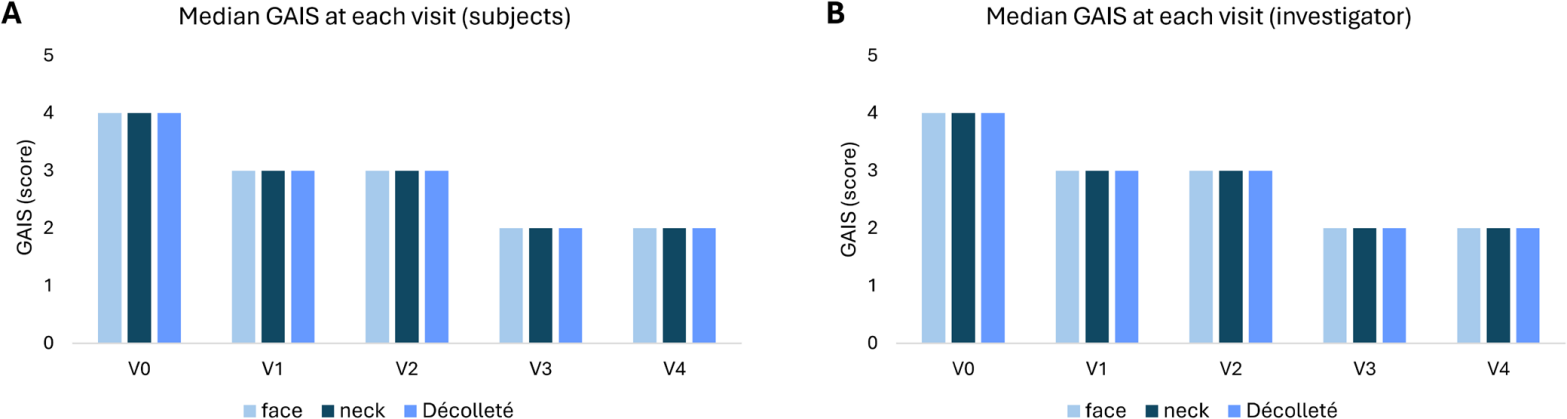
Aesthetic improvements assessed by subjects (A) and investigator (B) over time. GAIS, Global Aesthetic Improvement Scale.

At baseline, skin appearance was documented, and a value of 4 was arbitrarily and uniformly assigned to all treated areas according to the GAIS evaluation system, serving as the reference score. Changes from this initial value were subsequently evaluated at each follow‐up visit. Following the administration of the first and second injections, a median GAIS score of 3 (“improved”) was recorded across all treated areas, as assessed by both the participants and the investigator, indicating a 1‐point improvement compared with the baseline score. Further significant improvements were observed 2 months after the end of treatment (V3) in all areas under examination, reaching a value of 2 (“much improved”). This value remained stable even 4 months after treatment (V4); all differences from baseline were statistically significant (Figure 2; Tables S3 and S4).

These findings confirm that the primary objective was achieved, with statistically significant improvements in GAIS score between V0 and V4, showing a median difference of 2 points in all treated areas, as assessed by both subjects and the investigator.

**FIGURE 3:**
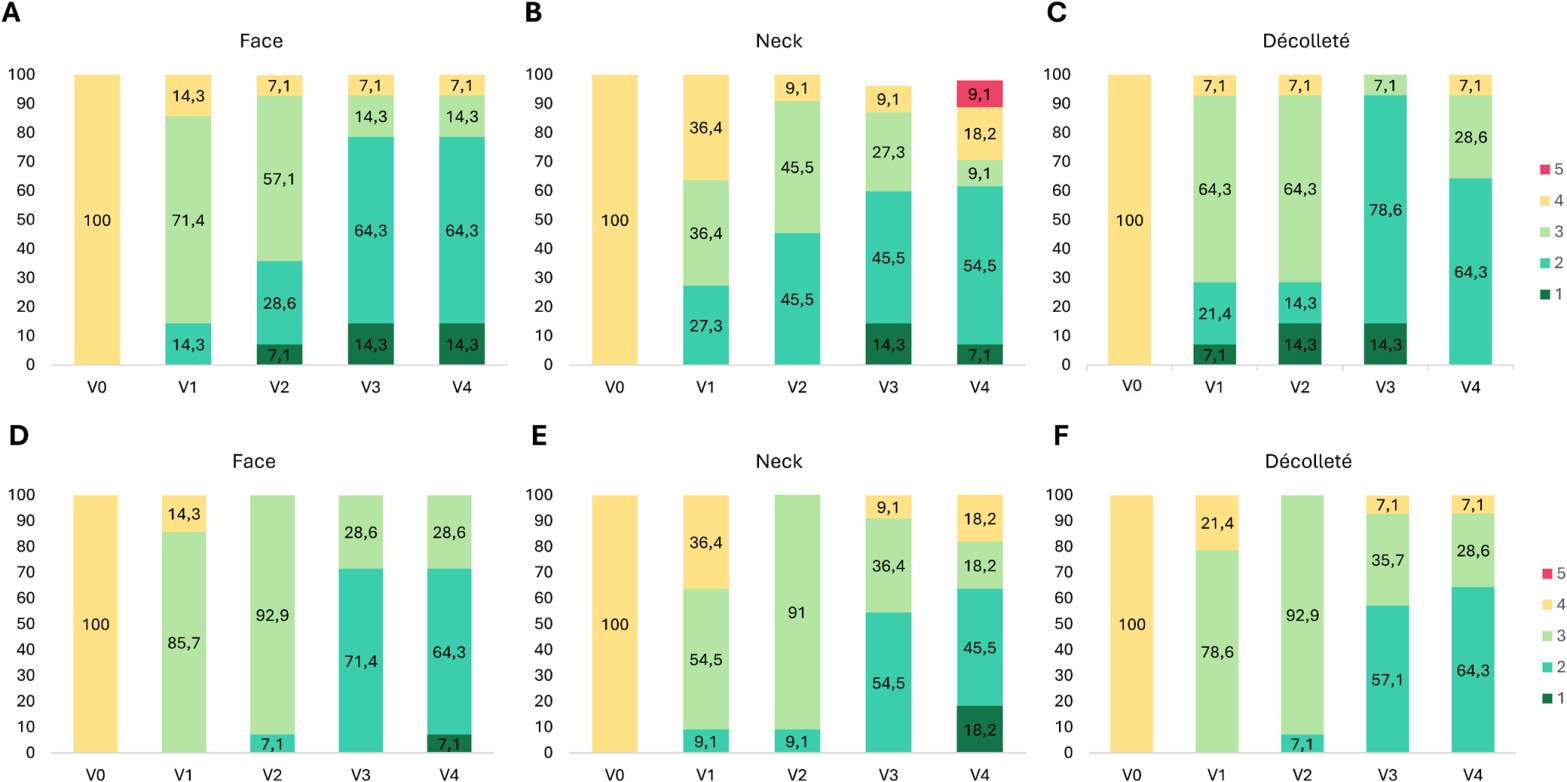
GAIS score distribution at each visit – participants (A–C) and investigator assessments (D–F).

Despite similar median GAIS, a closer examination of score distribution revealed differences according to the treated body area (Figure 3; Table S5). Moreover, a slight discrepancy between the scores assigned by the investigator and those reported by the participants was observed. Specifically, investigator‐assigned scores were more consistent, whereas participant‐reported evaluations exhibited greater variability (Table S6).

Patient evaluations revealed a general improvement in the condition of the skin but with different score distribution in the treated areas. In the facial region, a substantial proportion shifted from a GAIS score of 3 (“improved”) at V2 to 2 (“much improved”) at V3. This distribution remained stable at both V3 and V4, with 64.3% reporting GAIS = 2 and 14.3% GAIS = 1 (“very much improved” skin condition).

In the neck area, the proportion with a GAIS score of 4 (“no change”) decreased markedly between the first and second treatments. At V3, the majority (59.8%) reported a great improvement (GAIS 1 and 2), with 14.3% classified as GAIS 1. At V4, only one area (9.1%) reported a deterioration in skin condition (GAIS = 5), while the vast majority (70.7%) demonstrated a GAIS score ≤ 3.

A positive trend was also observed in the décolleté area, where more than 90% of ratings were classified as “much improved” or “very much improved” (GAIS scores of 2 and 1, respectively) at V3. The amelioration was sustained over time, with a slight reduction in the score (GAIS 2 decreasing from 78.6% at V3 to 64.3% at V4).

Similarly, investigator‐assigned scores revealed a general improvement but with a consistent GAIS score in all treated areas. For the facial area, at V1 and V2, most evaluations converged on a GAIS score of 3 (“improved”). Notably, the investigator reported no cases of worsening at V3 and V4, with 7.1% classified as “very much improved” at V4 (GAIS 1; Figure 3D).

Assessments of the neck region showed comparable trends between participants and the investigator, with the exception of V2, where investigator ratings displayed reduced variability across cases (Figure 3E).

In the décolleté area, at V1, 78.6% of cases were rated as “improved”, rising to 92.9% at V2. As illustrated in Figure 3F, the proportion of cases rated as “very much improved” increased progressively at V3 and V4.

**FIGURE 4:**
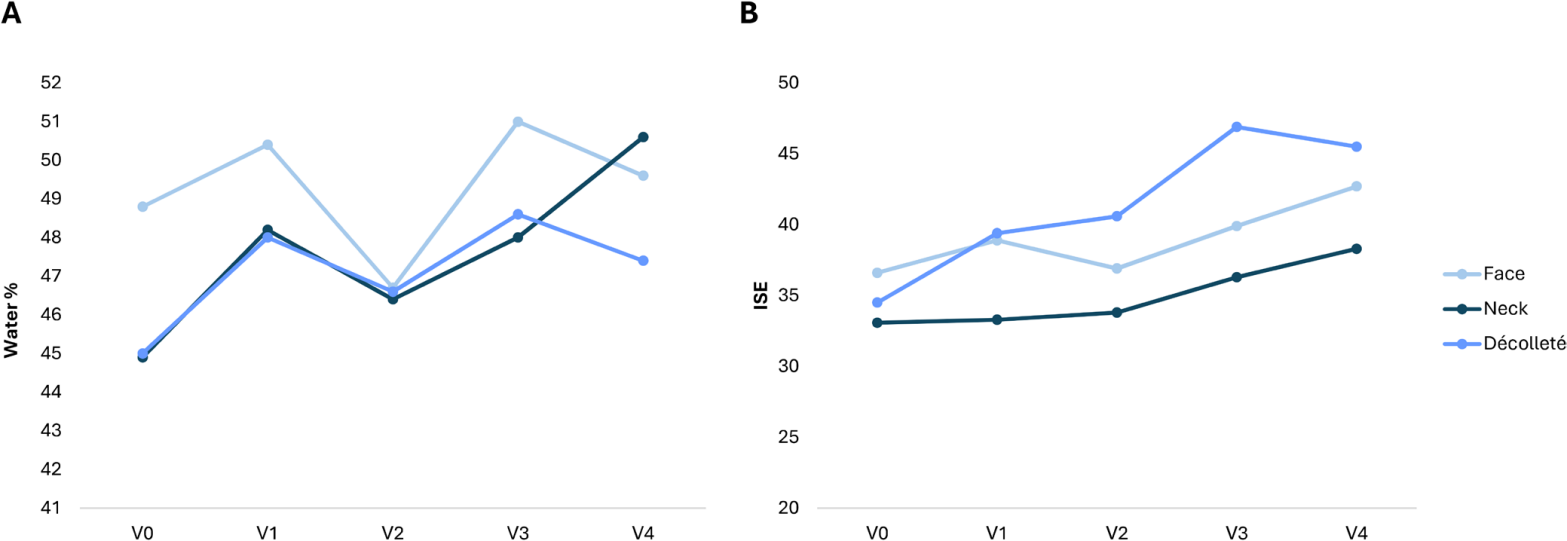
Changes in mean skin hydration (A) and elasticity (B) over time. ISE, instant skin elasticity.

Skin hydration status was assessed at all study visits (Figure 4A; Table S7). All three treated areas showed an overall increase in mean skin water content over time, with the neck exhibiting the greatest magnitude of improvement, although minor fluctuations were observed throughout the study period.

At V1, following the first injection, hydration levels in the superficial skin layer increased in a comparable manner across all treated areas. Facial hydration increased from 48.8 ± 5.9 at baseline to 50.4 ± 6.9 at V1. Similarly, neck and décolletage hydration increased from 44.9 and 45.0 at baseline to 48.2 and 48.0 at V1. At V2, a slight decline was observed in all treated areas, and a subsequent peak was recorded at V3. By the final assessment (V4), the hydration content of the skin in the neck region had increased to 50.6 ± 7.3, while hydration levels in the facial and décolleté regions had decreased slightly towards stabilization. The post hoc analysis revealed a statistically significant change between V4 and V0 in the neck region (*p* = 0.0131), while changes in the face and décolleté were not significant (*p* = 0.5580 and *p* = 0.4120, respectively).

Consistent with these findings, the ISE index demonstrated a progressive improvement in skin elasticity across all treated areas over time (Figure 4B; Table S8). The ElastiMeter (Delfin Technologies Ltd) tool provides an ISE (N/m) value, which indicates the stiffness of the skin in relation to the applied deformation. Consequently, higher values indicate greater resistance to compression. The neck region exhibited the lowest elasticity values at baseline (33.1 ± 3.7 N/m) with values increasing to 36.3 ± 5.8 N/m and 38.3 ± 5.6 N/m at 2 and 4 months after the last injection, respectively. In contrast, the décolleté showed the most pronounced enhancement in elasticity, with an increase of nearly 10 units between baseline and the final assessment. Face region had the highest elasticity values at baseline (mean 36.6 ± 6.1 N/m), with a peak observed at V2 and a sustained elasticity level above 40.0 N/m maintained at the final follow‐up. The post hoc analysis revealed statistically significant changes between V4 and V0 in all treated areas: face (*p* = 0.0477), neck (*p* = 0.0257), and décolleté (*p* = 0.0003).

Skin turgor recorded median values ranging from 2 to 4 throughout the study period; the neck region showed the greatest stability, maintaining a median score of 3 at all study visits (Table S9), the facial area showed a stable score of 3 from V0 through V2, with an improvement to a median score of 4 at V3, while the décolleté exhibited a lower baseline median value of 2 which increased to 3 from V1 to V3, and further increased to a median score of 4 at the final visit.

Pain intensity following each injection was generally reported as moderate, with higher pain levels observed in the face and neck, while pain in the décolleté area was typically mild. Of the three injections, the second was perceived as the most painful. However, for all participants, the duration of pain associated with each injection was less than 5 min. The final injection was associated with lower pain intensity across all treated areas (Table S10).

Patient‐reported outcomes indicated a high level of satisfaction with the treatment. Across all treated areas, satisfaction scores ranged from 4 to 5 at both V3 and V4 (Table S11).

### 3.3 Safety Outcomes

The safety analysis set included 28 participants, of whom 6 experienced at least one AE. A total of 10 AEs were reported. One case of herpes zoster, unrelated to the product, was classified as severe and led to study discontinuation. All other reported events were mild and resolved without consequences, including two cases of headache, one case of contusion, and one case of dizziness. The contusion was considered related to the intervention.

Overall, 25 subjects completed the study.

## 4 Discussion

Skin condition and aging can have an important social impact, influencing self‐esteem, self‐perception, and judgments of attractiveness [15, 16]. Despite growing public interest in rejuvenation, the literature on aesthetic treatments of the neck and lower face remains limited, and this study aims to contribute additional evidence to this field.

The utilization of PN HPT as a dermal filler in aesthetic medicine is a promising field, offering high levels of biocompatibility and tolerability, as well as minimal invasiveness. Several studies have demonstrated their effectiveness in skin rejuvenation both over the face and other body areas [13, 14, 17, 18, 19, 20, 21]. The GAIS score is a commonly used performance endpoint in this context, with assessments performed by both participants and investigators. In the present study, 28 subjects underwent dermal injections of a PN HPT‐based filler to enhance skin hydration and achieve aesthetic improvement of the face, neck, or décolleté, with evaluations performed up to 4 months after treatment. After three PN HPT injections administered at 2‐week intervals, skin appearance significantly improved in all treated areas, with a median difference of 2 points in the GAIS score. Although median GAIS values were similar across treated areas, the score distribution revealed greater variability across visits and anatomical sites. The face and décolleté areas achieved higher improvement scores than the neck, with a slight advantage for the face, particularly at the last follow‐up. However, the most marked improvement occurred between the second and third treatment sessions, with results maintained thereafter. At the end of the study, the neck showed higher variability in outcomes within the cohort, with 7.1% of participants considered their neck condition “very much improved”, whereas a higher proportion was reported by the clinician (18.2%), suggesting a lower self‐perception of improvement. For this reason, self‐assessment and patient satisfaction are increasingly important outcomes in clinical practice, especially for the neck area that is widely regarded as among the most challenging to assess and treat due to its complex anatomy and the continuous movements in daily activities [22].

These findings are further reinforced by the research of Eliana et al., which evaluated the efficacy and safety of a PN HPT‐based dermal filler in 106 subjects undergoing treatment of the face, neck, or décolleté. The study reported the highest percentage of patients achieving excellent results in the facial area compared with those treated with intradermal injections in the neck and décolleté [9].

PN HPT, long DNA fragments extracted from fish, are able to attract and bind water molecules, which are slowly and progressively released in situ following physiological enzymatic degradation. This contributes to improving skin hydration and supports steady self‐repair, normalizing the physiological dermal environment [17, 19]. Moreover, polynucleotides also demonstrated superior efficacy in improving skin roughness and reducing pore size compared to HA [16]. In this study, a trend towards improvement in both skin hydration and turgor over time was observed. Water content showed a steady increase after the first injection across all treated areas, followed by a decline at V2 and a subsequent increase thereafter. While the face exhibited the highest baseline water percentage (48.8%), this remained relatively consistent, with a final value of 49.6%. In contrast, PN HPT exhibited a more marked hydrating effect on the neck, ranging from 44.9% at the baseline to 50.6% at V4 (*p* = 0.0131).

In line with this observation, skin elasticity demonstrated progressive enhancement even after the conclusion of the treatment and the décolleté region was the one exhibiting the most pronounced change between V0 and V4, with an initial value of 34.5 N/m and a final value of 45.5 N/m (*p* = 0.0257). The face and neck regions demonstrated lower mean values, yet they exhibited a significant improvement from baseline of more than 5 N/m. This is of particular interest given that the skin on the décolleté and neck usually ages more severely than the skin on the face [23]. The décolleté is also considered a particularly vulnerable anatomical region due to its thinner skin, which makes it more susceptible to photodamage and wrinkling from sun exposure [24]. Indeed, the turgor of the décolleté region had the lowest baseline assessment in comparison with other areas.

Lim et al. investigated the effects of PN HPT‐based injections on skin tone evenness, skin surface evenness, skin firmness, and skin glow. They demonstrated meaningful improvements and a favorable safety profile, with no late‐onset adverse events. The benefits observed were sustained for a period of up to 6 months, and the authors attributed this to the capacity of PN HPT to facilitate fibroblast activity by enhancing the dermal environment [25]. These observations are particularly relevant within the ongoing discussion on the durability of injectable skin‐rejuvenation treatments, which may be influenced by manufacturing technology, tissue mobility, and anatomical factors. HA fillers are well known for their rapid effects, which are mainly related to their hydrating and space‐filling properties, while PN HPT is thought to act through a temporary support for tissue restoration [26]. This is reflected in the different timing of perceived benefits: although HA‐based treatments may induce earlier improvements, polynucleotides‐based treatments appear to maintain progressive improvement over time, potentially resulting in longer‐lasting effects [16].

Beside the performance evaluation, patient satisfaction—a significant factor in determining the success of treatment—was also confirmed. The present study examined patient satisfaction following a 2‐ and 4‐month period after the last injection. The results yielded a high degree of satisfaction, with a median score ranging between 4 and 5 on a scale of 1–5. Moreover, the investigational device demonstrated a satisfactory safety profile with only one case of mild contusion related to the intervention, which resolved spontaneously without repercussions, further supporting the feasibility of the procedure. The use of local anesthesia may have contributed to reducing discomfort during the procedure; however, pain perception associated with injectable treatments can vary according to individual patient sensitivity and the anatomical site treated. In this study, the pain experienced was moderate to severe in intensity, primarily in the face and neck region, with an increase in intensity following the second injection. In contrast, lower median values were recorded in the décolleté region. Although injection‐site pain is a common experience in cosmetic procedures, it is usually transient and resolves spontaneously [27], as was observed in this study. Therefore, the treatment under investigation exhibits the key attributes to be regarded as an optimal choice for both patients and clinicians, considering that the primary barriers to treatment selection have been reported to be pain, safety, and costs [28].

However, these findings should be considered in light of some limitations. The absence of a control group and the relatively small sample size may limit the generalizability and interpretability of the results. Furthermore, no stratification was performed according to baseline hydration levels, age, or lifestyle, which may offer a more comprehensive insight into the treatment outcomes according to different groups.

## 5 Conclusions

This study provides additional evidence supporting the safety and efficacy of naturally derived PN HPT for skin rejuvenation, suggesting sustained improvement in skin appearance and hydration even after a limited number of sessions, thereby reducing the need for more invasive procedures. These findings confirm the safety and efficacy of the device in this target group. However, future studies should include larger populations and stratified analyses to provide a more detailed picture of treatment responsiveness across different skin conditions and population groups.

## Author Contributions

All authors contributed to the study conception, data collection, analysis, and manuscript preparation. All authors reviewed and approved the final version of the manuscript.

## Funding

This prospective study was sponsored by Mastelli s.r.l. No funding or sponsorship was received by the authors for this study. Medical Writing assistance was provided by Clariscience S.r.l. (Padova, Italy) through a Mastelli s.r.l. unconditional grant. The authors of the publication are fully responsible for the contents and conclusions.

## Ethics Statement

The study was conducted in accordance with the Declaration of Helsinki and approved by the local Ethics Committee (Comitato Etico Territoriale Liguria) in October 2024. The study is registered on ClinicalTrial.gov (registration number: NCT06821854). All participants provided written informed consent for their involvement in the present investigation.

## Conflicts of Interest

The authors declare no conflicts of interest.

## Supporting information


**Figure S1:** Patients disposition.
**Table S1:** Inclusion and exclusion criteria.
**Table S2:** Treatment administration.
**Table S3:** GAIS assessed by patients at each visit (FAS population).
**Table S4:** GAIS assessed by investigator at each visit (FAS population).
**Table S5:** GAIS score distribution at each visit assessed by patients (FAS population).
**Table S6:** GAIS score distribution at each visit assessed by investigator (FAS population).
**Table S7:** Skin hydration at each visit (FAS population).
**Table S8:** Skin elasticity at each visit (FAS population).
**Table S9:** Skin turgor at each visit (FAS population).
**Table S10:** Pain assessment (FAS population).
**Table S11:** Patient satisfaction (FAS population).

## Data Availability

Data supporting the findings of this study are available within the article and its Supporting Information.
