# Supplementary material for: Polynucleotides HPT‐Based Dermal Filler for Skin Rejuvenation: A Prospective Clinical Investigation
Source: J Cosmet Dermatol. 2026 Aug 25;25(9):e71154. doi: 10.1111/jocd.71154 (PMC13507371; doi:10.1111/jocd.71154)
Supplement: Supplementary file 1 [file JOCD-25-e71154-s001.docx]

Table S1. Inclusion and exclusion criteria.

| Inclusion | Exclusion |
| --- | --- |
| Subject Informed consent form (ICF) signed | Other clinical conditions of the skin (i.e. rosacea, psoriasis, vitiligo, active eczema, severe scleroderma, severe acne and diagnosticated cancer with/without ongoing antitumor therapy) |
| Male or Female aged 18-70 years | Infectious or inflammatory processes near the area of intervention |
| Subjects desiring improvement of skin hydration in maximum 2 areas of the face, or 1 area of the face plus neck or décolleté | Presence of cutaneous disease on the tested area, as malformations and recurrent facial/labial herpes |
| Healthy skin | Presence of tendon, bone or muscular implants near the area of intervention |
| Accepting to not change their habits regarding food, physical activity, face/neck/décolleté cosmetics and cleansing products | Ongoing cutaneous allergies |
| Willingness to follow indications to minimize exposure of the treated area to too much sunlight or extreme cold, at least until the papules have completely subsided | Allergy or contraindications to device components |
| Skin phototype I-IV according to Fitzpatrick’s classification | Concomitant intake of anticoagulant or antiplatelet medications |
|  | Subjects who have not followed a washout period of 2 weeks from topical corticosteroids, antibiotics, benzoylperoxide, azelaic acid, hydroxy acids, topical retinoids |
|  | Immune system illnesses/disease |
|  | Uncontrolled diabetes mellitus or uncontrolled systemic diseases (endocrine, hepatic renal, cardiac, pulmonary, neurological disorder) |
|  | Treatment with substances which act on blood fluidity (e.g. Aspirin, NSAIDs, Vitamin E), or drugs able to influence the test results in the investigator opinion, within 5 days prior to study inclusion |
|  | Known drug and/or alcohol abuse |
|  | Mental incapacity that precludes adequate understanding or cooperation |
|  | Any previous permanent and non-permanent cutaneous treatment for aesthetic correction of the treated area within 6 months prior to study inclusion |
|  | Pregnancy or breastfeeding |
|  | Participation in another investigational study within 1 month prior to study inclusion |

Table S2. Treatment administration

|  | V0 | V1 | V2 |
| --- | --- | --- | --- |
| Combined treated areas | | | |
| Face + Neck | 8 (28.6) | 8 (30.7) | 7 (26.9) |
| Face + Décolleté | 7 (25.0) | 7 (26.9) | 7 (26.9) |
| Single treated areas | | | |
| Face | 0 | 0 | 0 |
| Neck | 6 (21.4) | 4 (15.4) | 4 (15.4) |
| Décolleté | 7 (25.0) | 7 (26.9) | 7 (26.9) |
| Overall treated areas | | | |
| Face | 15 (53.6) | 15 (57.8) | 14 (53.8) |
| Neck | 14 (50.0) | 12 (46.1) | 11 (42.3) |
| Décolleté | 14 (50.0) | 14 (53.8) | 14 (53.8) |

*Data are reported as n (%). Percentages are calculated based on the total number of subjects at each visit.*

Table S3. GAIS assessed by patients at each visit (FAS population)

| Area | Value (score) | V0 | V1 | ∆ V0-V1 | p-value | V2 | ∆ V0-V2 | p-value | V3 | ∆ V0-V3 | p-value | V4 | ∆ V0-V4 | p-value |
| --- | --- | --- | --- | --- | --- | --- | --- | --- | --- | --- | --- | --- | --- | --- |
| Face | median | 4 | 3 | 1 | **0.0005** | 3 | 1 | **0.0002** | 2 | 2 | **0.0002** | 2 | 2 | **0.0002** |
|  | Q1;Q3 | 4;4 | 3;3 | 1;1 |  | 2;3 | 1;2 |  | 2;2 | 2;2 |  | 2;2 | 2;2 |  |
|  | Min;Max | 4;4 | 2;4 | 0;2 |  | 1;4 | 0;3 |  | 1;4 | 0;3 |  | 1;4 | 0;3 |  |
|  | missing | 0 | 0 | 0 |  | 1 | 1 |  | 1 | 1 |  | 1 | 1 |  |
| Neck | median | 4 | 3 | 1 | **0.0078** | 3 | 1 | **0.002** | 2 | 2 | **0.002** | 2 | 2 | **0.0117** |
|  | Q1;Q3 | 4;4 | 2.5;4 | 0;1.5 |  | 2;3 | 1;2 |  | 2;3 | 1;2 |  | 2;4 | 0;2 |  |
|  | Min;Max | 4;4 | 2;4 | 0;2 |  | 2;4 | 0;2 |  | 1;4 | 0;3 |  | 1;5 | -1;3 |  |
|  | missing | 0 | 0 | 0 |  | 1 | 1 |  | 1 | 1 |  | 1 | 1 |  |
| Décolleté | median | 4 | 3 | 1 | **0.0002** | 3 | 1 | **0.0002** | 2 | 2 | **0.0001** | 2 | 2 | **0.0002** |
|  | Q1;Q3 | 4;4 | 2;3 | 1;2 |  | 2;3 | 1;2 |  | 2;2 | 2;2 |  | 2;3 | 1;2 |  |
|  | Min;Max | 4;4 | 1;4 | 0;3 |  | 1;4 | 0;3 |  | 1;3 | 1;3 |  | 2;4 | 0;2 |  |
|  | missing | 0 | 0 | 0 |  | 0 | 0 |  | 0 | 0 |  | 0 | 0 |  |

Table S4. GAIS assessed by investigator at each visit (FAS population)

| Area | Value (score) | V0 | V1 | ∆ V0-V1 | p-value | V2 | ∆ V0-V2 | p-value | V3 | ∆ V0-V3 | p-value | V4 | ∆ V0-V4 | p-value |
| --- | --- | --- | --- | --- | --- | --- | --- | --- | --- | --- | --- | --- | --- | --- |
| Face | median | 4 | 3 | 1 | **0.0005** | 3 | 1 | **0.0001** | 2 | 2 | **0.0001** | 2 | 2 | **0.0001** |
|  | Q1;Q3 | 4;4 | 3;3 | 1;1 |  | 3;3 | 1;1 |  | 2;3 | 1;2 |  | 2;3 | 1;2 |  |
|  | Min;Max | 4;4 | 3;4 | 0;1 |  | 2;3 | 1;2 |  | 2;3 | 1;2 |  | 1;3 | 1;3 |  |
|  | missing | 0 | 0 | 0 |  | 1 | 1 |  | 1 | 1 |  | 1 | 1 |  |
| Neck | median | 4 | 3 | 1 | **0.0078** | 3 | 1 | **0.001** | 2 | 2 | **0.002** | 2 | 2 | **0.0039** |
|  | Q1;Q3 | 4;4 | 3;4 | 0;1 |  | 3;3 | 1;1 |  | 2;3 | 1;2 |  | 2;3 | 1;2 |  |
|  | Min;Max | 4;4 | 2;4 | 0;2 |  | 2;3 | 1;2 |  | 2;4 | 0;2 |  | 1;4 | 0;3 |  |
|  | missing | 0 | 0 | 0 |  | 1 | 1 |  | 1 | 1 |  | 1 | 1 |  |
| Décolleté | median | 4 | 3 | 1 | **0.001** | 3 | 1 | **0.0001** | 2 | 2 | **0.002** | 2 | 2 | **0.0002** |
|  | Q1;Q3 | 4;4 | 3;3 | 1;1 |  | 3;3 | 1;1 |  | 2;3 | 1;2 |  | 2;3 | 1;2 |  |
|  | Min;Max | 4;4 | 3;4 | 0;1 |  | 2;3 | 1;2 |  | 2;4 | 0;2 |  | 2;4 | 0;2 |  |
|  | missing | 0 | 0 | 0 |  | 0 | 0 |  | 0 | 0 |  | 0 | 0 |  |

Table S5. GAIS score distribution at each visit assessed by patients (FAS population)

| Area | Score | V1 | V2 | V3 | V4 |
| --- | --- | --- | --- | --- | --- |
| Face n=14 | 1 | 0 | 1 (7.1) | 2 (14.3) | 2 (14.3) |
|  | 2 | 2 (14.3) | 4 (28.6) | 9 (64.3) | 9 (64.3) |
|  | 3 | 10 (71.4) | 8 (57.1) | 2 (14.3) | 2 (14.3) |
|  | 4 | 2 (14.3) | 1 (7.1) | 1 (7.1) | 1 (7.1) |
|  | 5 | 0 | 0 | 0 | 0 |
| Neck n=11 | 1 | 0 | 0 | 2 (14.3) | 1 (7.1) |
|  | 2 | 3 (27.3) | 5 (45.5) | 5 (45.5) | 6 (54.5) |
|  | 3 | 4 (36.4) | 5 (45.5) | 3 (27.3) | 1 (9.1) |
|  | 4 | 4 (36.4) | 1 (9.1) | 1 (9.1) | 2 (18.2) |
|  | 5 | 0 | 0 | 0 | 1 (9.1) |
| Décolleté n=14 | 1 | 1 (7.1) | 2 (14.3) | 2 (14.3) | 0 |
|  | 2 | 3 (21.4) | 2 (14.3) | 11 (78.6) | 9 (64.3) |
|  | 3 | 9 (64.3) | 9 (64.3) | 1 (7.1) | 4 (28.6) |
|  | 4 | 1 (7.1) | 1 (7.1) | 0 | 1 (7.1) |
|  | 5 | 0 | 0 | 0 | 0 |

*Values are presented as n (%)*

Table S6. GAIS score distribution at each visit assessed by investigator (FAS population)

| Area | Score | V1 | V2 | V3 | V4 |
| --- | --- | --- | --- | --- | --- |
| Face n=14 | 1 | 0 | 0 | 0 | 1 (7.1) |
|  | 2 | 0 | 1 (7.1) | 10 (71.4) | 9 (64.3) |
|  | 3 | 12 (85.7) | 13 (92.9) | 4 (28.6) | 4 (28.6) |
|  | 4 | 2 (14.3) | 0 | 0 | 0 |
|  | 5 | 0 | 0 | 0 | 0 |
| Neck n=11 | 1 | 0 | 0 | 0 | 2 (18.2) |
|  | 2 | 1 (9.1) | 1 (9.1) | 6 (54.5) | 5 (45.5) |
|  | 3 | 6 (54.5) | 10 (91.0) | 4 (36.4) | 2 (18.2) |
|  | 4 | 4 (36.4) | 0 | 1 (9.1) | 2 (18.2) |
|  | 5 | 0 | 0 | 0 | 0 |
| Décolleté n=14 | 1 | 0 | 0 | 0 | 0 |
|  | 2 | 0 | 1 (7.1) | 8 (57.1) | 9 (64.3) |
|  | 3 | 11 (78.6) | 13 (92.9) | 5 (35.7) | 4 (28.6) |
|  | 4 | 3 (21.4) | 0 | 1 (7.1) | 1 (7.1) |
|  | 5 | 0 | 0 | 0 | 0 |

*Values are presented as n (%)*

Table S7. Skin hydration at each visit (FAS population)

| Area | Value (%) | V0 | V1 | V2 | V3 | V4 |
| --- | --- | --- | --- | --- | --- | --- |
| Face | mean | 48.8 | 50.4 | 46.7 | 51.0 | 49.6 |
|  | SD | 5.9 | 6.9 | 7.3 | 6.6 | 6.2 |
|  | Min;Max | 36.0; 57.0 | 39.0; 61.0 | 31.0; 56.0 | 40.0; 63.0 | 40.0; 62.0 |
|  | missing | 0 | 0 | 1 | 1 | 1 |
| Neck | mean | 44.9 | 48.2 | 46.4 | 48.0 | 50.6 |
|  | SD | 7.2 | 8.0 | 9.2 | 6.2 | 7.3 |
|  | Min;Max | 31.0; 56.0 | 31.0; 62.0 | 29.0; 63.0 | 38.0; 56.0 | 41.0; 62.0 |
|  | missing | 0 | 0 | 1 | 1 | 1 |
| Décolleté | mean | 45.0 | 48.0 | 46.6 | 48.6 | 47.4 |
|  | SD | 9.3 | 7.0 | 7.5 | 5.3 | 4.1 |
|  | Min;Max | 30.0; 60.0 | 36.0; 57.0 | 27.0; 54.0 | 37.0; 55.0 | 42.0; 56.0 |
|  | missing | 0 | 0 | 0 | 0 | 0 |

Table S8. Skin elasticity at each visit (FAS population)

| Area | Value (N/m) | V0 | V1 | V2 | V3 | V4 |
| --- | --- | --- | --- | --- | --- | --- |
| Face | mean | 36.6 | 38.9 | 36.9 | 39.9 | 42.7 |
|  | SD | 6.1 | 8.6 | 7.8 | 6.5 | 8.1 |
|  | Min;Max | 30.0; 49.0 | 29.0; 63.0 | 29.0; 56.0 | 31.0; 52.0 | 31.0; 60.0 |
|  | missing | 0 | 0 | 1 | 1 | 1 |
| Neck | mean | 33.1 | 33.3 | 33.8 | 36.3 | 38.3 |
|  | SD | 3.7 | 4.1 | 5.4 | 5.8 | 5.6 |
|  | Min;Max | 29.0; 42.0 | 29.0; 41.0 | 29.0; 48.0 | 27.0; 50.0 | 32.0; 48.0 |
|  | missing | 0 | 0 | 1 | 1 | 1 |
| Décolleté | mean | 34.5 | 39.4 | 40.6 | 46.9 | 45.5 |
|  | SD | 4.7 | 5.7 | 9.4 | 11.5 | 7.5 |
|  | Min;Max | 29.0; 48.0 | 32.0; 53.0 | 29.0; 63.0 | 35.0; 73.0 | 35.0; 64.0 |
|  | missing | 0 | 0 | 0 | 0 | 0 |

Table S9. Skin turgor at each visit (FAS population)

| Area | Value (score) | V0 | V1 | V2 | V3 | V4 |
| --- | --- | --- | --- | --- | --- | --- |
| Face | median | 3 | 3 | 3 | 4 | 4 |
|  | Q1;Q3 | 2;3 | 3;3 | 3;3 | 3;4 | 4;4 |
|  | Min;Max | 2;3 | 2;3 | 2;4 | 3;4 | 3;4 |
|  | missing | 0 | 0 | 1 | 1 | 1 |
| Neck | median | 3 | 3 | 3 | 3 | 3 |
|  | Q1;Q3 | 2;3 | 3;3 | 3;4 | 3;4 | 3;4 |
|  | Min;Max | 2;3 | 2;3 | 3;4 | 2;4 | 2;5 |
|  | missing | 0 | 0 | 1 | 1 | 1 |
| Décolleté | median | 2 | 3 | 3 | 3 | 4 |
|  | Q1;Q3 | 2;3 | 3;3 | 3;3 | 3;4 | 3;4 |
|  | Min;Max | 2;3 | 2;3 | 2;4 | 2;4 | 3;5 |
|  | missing | 0 | 0 | 0 | 0 | 0 |

Table S10. Pain assessment (FAS population)

| Area | Value (score) | V0 | V1 | V2 |
| --- | --- | --- | --- | --- |
| Face | median | 7 | 8 | 7 |
|  | Q1;Q3 | 6;9 | 7;9 | 5;8 |
|  | Min;Max | 5;9 | 5;10 | 2;10 |
|  | missing | 0 | 0 | 1 |
| Neck | median | 7.5 | 7.5 | 6 |
|  | Q1;Q3 | 3,5; 9 | 3;9 | 2;8 |
|  | Min;Max | 2;9 | 2;10 | 1;10 |
|  | missing | 0 | 0 | 1 |
| Décolleté | median | 5.5 | 7 | 5 |
|  | Q1;Q3 | 4;7 | 2;8 | 2;7 |
|  | Min;Max | 1;9 | 1;9 | 0;10 |
|  | missing | 0 | 0 | 0 |

Table S11. Patient satisfaction (FAS population)

| Area | Value (score) | V3 | V4 |
| --- | --- | --- | --- |
| Face | median | 5 | 4 |
|  | Q1;Q3 | 4;5 | 4;4 |
|  | Min;Max | 2;5 | 2;5 |
|  | missing | 1 | 1 |
| Neck | median | 4 | 4 |
|  | Q1;Q3 | 3;5 | 4;4 |
|  | Min;Max | 2;5 | 2;5 |
|  | missing | 1 | 1 |
| Décolleté | median | 5 | 4 |
|  | Q1;Q3 | 4;5 | 4;5 |
|  | Min;Max | 4;5 | 4;5 |
|  | missing | 0 | 0 |

Figure S1. Patients disposition.

Abbreviations: SAS, Safety Analysis Set; FAS, Full Analysis Set; PPS, Per Protocol Set.
